# Assessing the content validity of the Manchester–Oxford Foot Questionnaire in surgically treated ankle fracture patients: a qualitative study

**DOI:** 10.1186/s13018-023-04418-9

**Published:** 2023-12-08

**Authors:** Michael Q. Nguyen, Anders Broström, Marjolein M. Iversen, Knut Harboe, Aksel Paulsen

**Affiliations:** 1 Department of Quality and Health Technology, Faculty of Health Sciences, University of Stavanger, Stavanger, Norway; 2 Department of Orthopedic Surgery, Stavanger University Hospital, Helse Stavanger HF, Stavanger, Norway; 3 Department of Orthopedic Surgery, The Fracture Registry of Western Norway, Stavanger University Hospital, Helse Vest RHF, Stavanger, Norway; 4 School of Health and Welfare, Jönköping University, Jönköping, Sweden; 5 Department of Clinical Neurophysiology, Linköping University Hospital, Linköping, Sweden; 6 Department of Health and Caring Sciences, Faculty of Health and Social Sciences, Western Norway University of Applied Sciences, Bergen, Norway; 7 Centre on Patient-Reported Outcomes, Department of Research and Development, Haukeland University Hospital, Helse Bergen HF, Bergen, Norway; 8 Department of Clinical Medicine, Faculty of Medicine, University of Bergen, Bergen, Norway; 9 Department of Anesthesia, Stavanger University Hospital, Helse Stavanger HF, Stavanger, Norway; 10 Department of Public Health, Faculty of Health Sciences, University of Stavanger, Stavanger, Norway

**Keywords:** Ankle fractures, Patient-reported outcome measures, Validation study, Qualitative research, Content validity

## Abstract

**Background:**

Roughly 10% of fractures in adults are ankle fractures. These injuries are found in both sexes and present with different fracture characteristics. The treatment varies with the patients’ biology and fracture type, and the goals are to restore stability, prevent pain and maintain ankle function. Clinicians generally use outcomes like assessment of radiography, pain level, or function. The use of patient-reported outcome measures is increasing, and the Manchester–Oxford Foot Questionnaire (MOXFQ) has been shown to have good measurement properties when validated in patients with foot and ankle disorders. However, the instrument has not been validated for ankle fracture patients. This study aims to assess the content validity of the items in MOXFQ in surgically treated ankle fracture patients.

**Methods:**

A qualitative deductive design was used to investigate patients’ response process of the MOXFQ. Individual interviews were conducted using cognitive interviewing based on the theoretical framework of the 4-step model by Tourangeau. Adult patients that were surgically treated for an ankle fracture between four weeks and 18 months were purposively sampled, and interviews followed a semi-structured interview guide. The predetermined categories were *comprehension*, *retrieval*, *judgement*, and *response*.

**Results:**

Seventeen respondents (65% females) were interviewed. Respondents’ age ranged from 27 to 76 years. Some of the respondents in the early recovery phase were limited by post-operative restrictions and did not find the items in the walking/standing domain relevant. Respondents that were allowed weight-bearing as tolerated (WBAT) were able to recall relevant information for most items. Respondents with time since surgery more than 12 months had less pain and remembered fewer relevant episodes in the recall period. Items in the social interaction domain contained ambiguous questions and were generally considered less important by respondents. The summary index score lacked important concepts in measuring overall quality of life.

**Conclusions:**

Pain was a central concept in the post-operative recovery of ankle fracture patients. The MOXFQ-subscales for pain and walking/standing had acceptable content validity in patients that were allowed WBAT. The social interaction-subscale and the summary index score had insufficient content validity for this patient population.

**Supplementary Information:**

The online version contains supplementary material available at 10.1186/s13018-023-04418-9.

## Background

Roughly 10% of all fractures in adults are due to ankle fractures [1]. Studies report incidence rates of 71/100,000 person years to as high as 187/100,000 person years [2, 3]. The incidence of ankle fractures is slightly higher in females. Young males suffer more ankle fractures when compared to older males, while incidence rates for females demonstrates a bimodal distribution with a peak in the second decade of life and an increased incidence after the fifth decade [4, 5].

The characteristics of ankle fractures are dependent on several factors, e.g., the mechanism of injury, the amount of energy and biological factors [6]. The primary treatment goal is the restoration of stability in the ankle joint, thereby preventing pain and maintaining ankle function, and also to reduce the risk of developing ankle joint arthrosis [7, 8]. Lateral malleolar fractures are the most common fracture type. Bi- and tri-malleolar are more common in the older female group compared to the older male group, likely associated with fragility fractures in the growing elderly population [4, 6, 9]. The treatment of fragility fractures is also more complex, in view of different surgical techniques, choice of implants and the patients’ biological factors. In the follow-up of ankle fracture patients, traditional outcome measures are used, e.g., X-rays, assessment of pain level, or range of motion. Nevertheless, the use of patient-reported outcome measures (PROMs) is growing in popularity, reflecting the desirability of its application in clinical practice [10], e.g., in the assessment and follow-up of patients [11]. This potentially provides the clinicians with unique and patient-specific information in the evaluation of the patient and treatment. PROMs are also used at the group level to monitor populations, inform decision-making, and assess quality of care [12, 13].

**Fig. 1:**
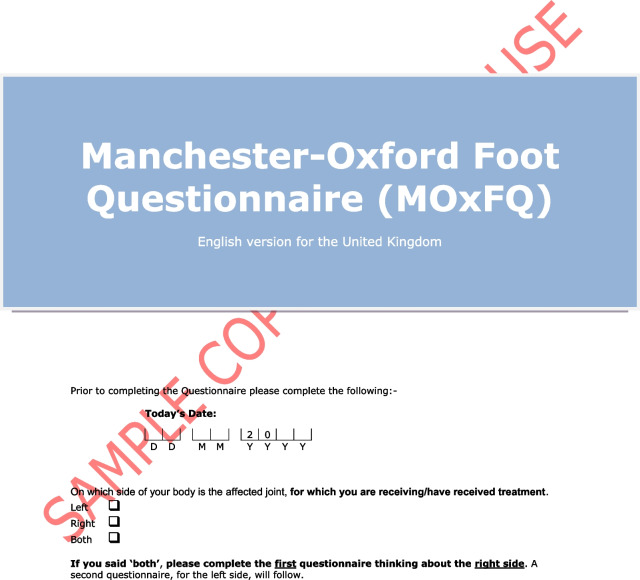

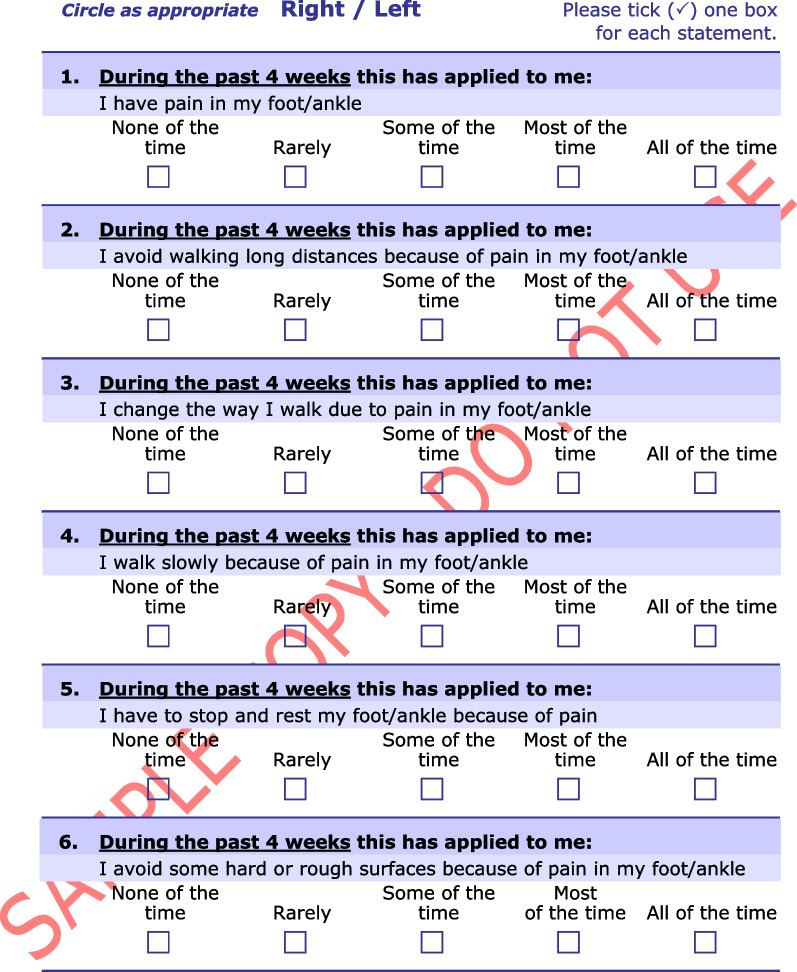

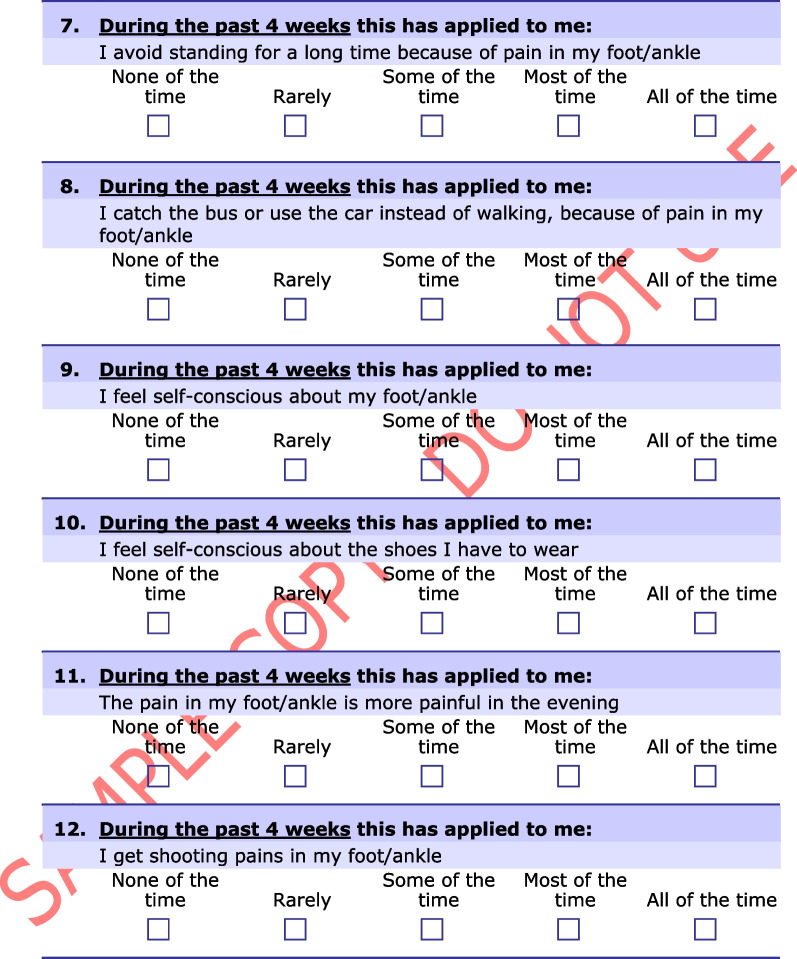

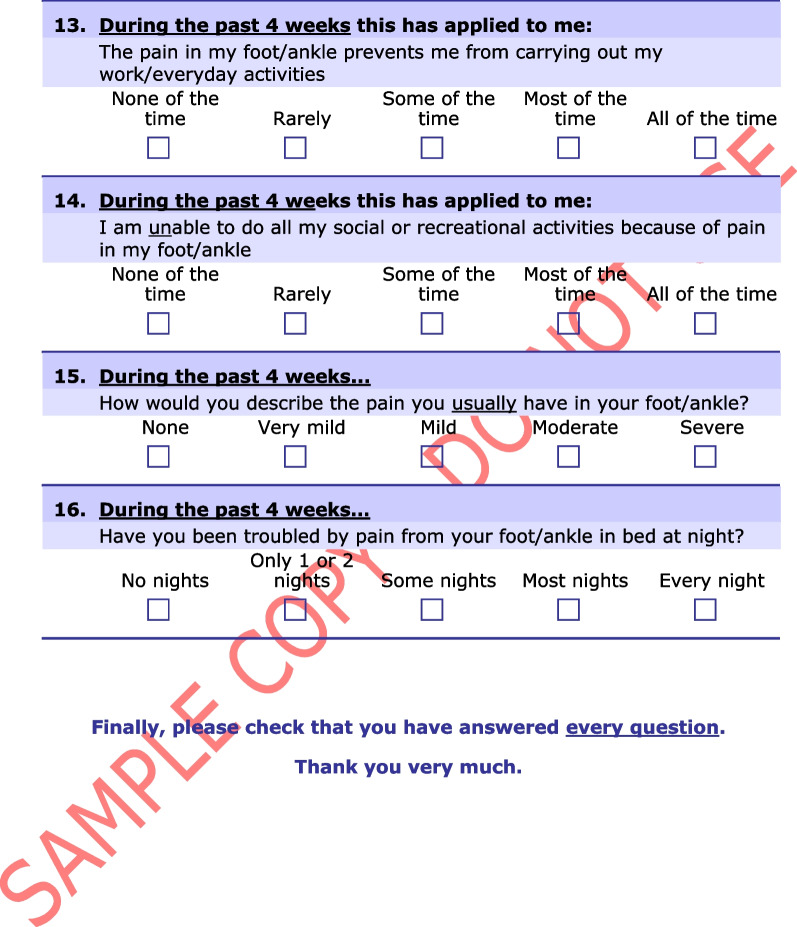
The Manchester–Oxford Foot Questionnaire. From “The MOXFQ patient-reported questionnaire: assessment of data quality, reliability and validity in relation to foot and ankle surgery”, by Dawson J, Boller I, Doll H, Lavis G, Sharp R, Cooke P, et al., The Foot, 2011;21(2):92–102. Any use of the MOxFQ in any language and for any purpose can only be made under licence from OUI and by contacting healthoutcomes@innovation.ox.ac.uk. © Oxford University Innovation Limited, 2006. All rights reserved. Reprinted with permission

The Manchester–Oxford Foot Questionnaire (MOXFQ) is a 16-item PROM developed to be used as an outcome measure of foot surgery [14]. The developers performed semi-structured interviews on patients scheduled for hallux valgus surgery, exploring problems relating to their foot condition. The instrument was later re-validated in patients undergoing surgery for different disorders of the foot and ankle (Fig. 1) [15]. In 2015, the Norwegian Foot and Ankle Society (NOFAF) advocated the use of the MOXFQ for patients with foot and ankle disorders [16]. In recent years, several studies have used the MOXFQ as a clinical outcome in various orthopedic foot and ankle conditions, including fractures [17–22]. A systematic review reported that MOXFQ had the overall highest ratings when validating measurement properties of PROMs used in patients with foot or ankle disease, although with a fundamental lack in the validation of its content validity [23]. Another systematic review demonstrated that the MOXFQ was the fourth most common multi-item PROM used as primary outcome measure in interventional trials for ankle fractures [24]. The most recent systematic review evaluating the evidence of measurement properties for PROMs used in patients with ankle fractures, revealed the absence of validation studies for the MOXFQ in this population [25]. However, when a PROM is applied to a new context of use, e.g., the use of existing measures for similar conditions, additional validation studies of the instrument’s measurement properties is warranted to ensure its validity and reliability. According to the Consensus-based standards for the selection of health measurement instruments (COSMIN), it is recommended to start with the most important measurement property, i.e., the content validity [12]. This includes examining the relevance, comprehensiveness and comprehensibility of the instrument in the new population [26]. Therefore, a validation study assessing the content validity of the MOXFQ in an ankle fracture patient population is justified.

PROMs are often used in a survey setting, as the instruments are intended to be completed independently by the respondents [27]. Already in early 1980s, survey methodologists and psychologist have tried to bridge the gap between the cognitive sciences and survey methodology through the project Cognitive Aspects Survey Methodology [28], in order to better understand the cognitive processes involved in the survey response. The validity evidence based on response processes, i.e., *evidence concerning the fit between the construct and the detailed nature of the performance or response actually engaged in by test takers* [29], can be improved by employing cognitive interviewing in the development and adaptation of survey measurements [30]. This study aims to assess the content validity of the MOXFQ in surgically treated ankle fracture patients by investigating the response processes.

## Methods

The Standards for Reporting Qualitative Research (SRQR) [31] and the Consolidated criteria for reporting qualitative studies (COREQ) [32] were used in the reporting of this article.

### Design and method description

**Fig. 2:**
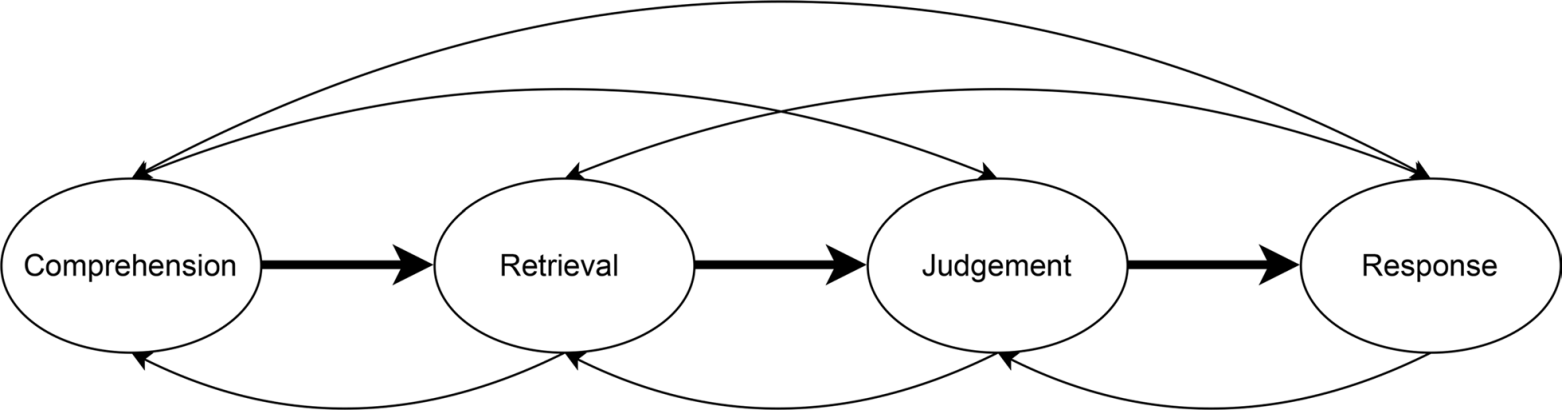
Tourangeau’s 4-step model. The figure illustrates the four cognitive operations that respondents undergo when replying to a survey [33–35]

A qualitative deductive design was used to investigate patients’ response processes of the MOXFQ. Individual interviews were conducted using cognitive interviewing based on the theoretical framework of the 4-step model by Tourangeau (Fig. 2), which addresses the stages respondents undergo when replying to survey questions [33–35]. The first step, the *comprehension*, focuses on how the respondent understands the question. Step two, the *retrieval of relevant information*, involves the information that the respondent needs to recall, and the strategy used to recall the information to answer the question. In step 3, labeled *judgement*, the cognitive effort used to evaluate relevant information to accurately answer the question is addressed. The last step, the *response*, is asking about the choices that the respondent makes when selecting a suitable response.

### The Manchester–Oxford Foot Questionnaire (MOXFQ)

The license to use the Norwegian version of the MOXFQ was obtained from Clinical Outcomes at Oxford University Innovation (Additional file 1). The translation and cross-cultural adaptation process has been performed by a third party and followed a minimum methodology with at least two forward translations and one backward translation. The instrument includes 16 items distributed over three domains: (1) the pain domain (five items); (2) the walking/standing domain (seven items); and (3) the social interaction domain (four items) (Table 1). The items have a recall period of four weeks and are intended to be completed with responses given on a 5-point Likert scale, where zero indicates least problems and four indicates greatest problems. A score for each domain is presented on a scale from 0 to 100 where a higher score denotes greater severity [14]. The scores reflect the impact of foot and ankle problems on the specific domains. An overall score, referred to as the index score, is calculated in the same matter, except the sum score is obtained from all 16 items [36]. The index score aims to measure the overall impact of foot and ankle problems on health-related quality of life (HRQOL).

**Table 1:** The division of the Manchester–Oxford Foot Questionnaire’s items into three domains

Domain	Item	Subject matter
Pain	1	Pain
	11	Evening pain
	12	Shooting pain
	15	Usual pain
	16	Night pain
Walking/standing	2	Avoid long distances due to pain
	3	Change the way of walking due to pain
	4	Walk slowly due to pain
	5	Stop and rest due to pain
	6	Avoid hard and rough surfaces due to pain
	7	Avoid standing for a long time due to pain
	8	Bus or car due to pain
Social interaction	9	Self-conscious about foot/ankle
	10	Self-conscious about footwear
	13	Limitation in work/everyday activities due to pain
	14	Limitations in social or recreational activities due to pain

### Participants and setting

Patients who were surgically treated at the Stavanger University Hospital for a unilateral ankle fracture classified as AO/OTA 44 [37] and at least 18 years of age were potential eligible candidates. The exclusion criteria were: less than 4 weeks or more than 18 months since surgery; other major concomitant injuries; patients with cognitive impairment or inability to give informed written consent; inability to speak, read or write fluently in Norwegian; major illnesses, other injuries or surgeries affecting impact of the ankle fracture on HRQOL 12 months prior or after the ankle fracture; other dominant musculoskeletal disorders. Purposive sampling [38] was conducted to ensure heterogeneity in the respondent group concerning age, sex, time since surgery, fracture characteristics, and pre-operative physical status assessed with the American Society of Anesthesiology (ASA) score [39] (Additional file 2). The post-operative routines for surgically treated ankle fracture patients often include partial weight bearing, and follow-up appointments at two and six weeks. Patients are usually allowed weight bearing as tolerated (WBAT) after the consultation at six weeks. The selection of respondents was stratified into three groups: (1) early-phase recovery, i.e., less than three months after surgery; (2) middle-phase recovery, i.e., from 3 to 12 months after surgery; and (3) late-phase recovery, i.e., more than 12 months after surgery. The interviews were conducted in a quiet, separate facility next to the hospital or in the respondents’ home, if preferred by the respondent.

### Data collection

A semi-structured interview-guide (Table 2) was constructed in conjunction with a research team consisting of clinical researchers with background in qualitative research, validation of PROMs, and fracture treatment. Two pilot interviews were conducted to test and accommodate the interview-guide, resulting in modest changes, e.g., improving the wording to augment the interview’s intent. These interviews were not included in the analyses. Some questions regarding retrieval and judgment of relevant episodes were rewritten or removed for increased clarity, e.g., that the relevance of each question was directed toward the respondent’s own experience. Minor adjustments to the order and wording of the questions asked were also implemented as the interviews were conducted and analyzed (“Generally, what are your thoughts on the relevance of these questions considering your ankle fracture?”). To capture all important aspects of the response process, the interviewer adjusted his approach during the data collection, especially with use of follow-up questions, such as “how did you proceed in choosing your response?”.

**Table 2:** Interview guide displaying questions and probes used to assess the MOXFQ

Areas of the MOXFQ assessed	Questions				Probes		
Face validity	In general, what are your thoughts on the questionnaire?						
Instructions	What are your thoughts on the instructions?	Can you describe any difficulties in understanding the instructions?			Is the language clear?	Are there any words or sentences you would like to change to make the instructions better?	
Items	What are your thoughts on this question?	Does the question capture problems that are, or have been relevant to you? If yes, please tell me more	What do you think the question is asking you?	What are your thoughts on the language used in the question?	Is something unclear or that can be misinterpreted?	Is the question worded in a way that can be offensive to you?	
Content of domains/instrument	Question [items in a domain] ask about [construct]. What other experiences did you have with [construct], that are not mentioned here?	What are your thoughts on other topics that are important to ask about concerning your ankle fracture, that are not mentioned here?					
Response options	What are your thoughts on the response options?	What do you think of when selecting a response option?	How do you assess the different response options?		How often is “never, rarely, some of the time, most of the time, all the time?” What about the last two questions?	Does the response options make sense?	
Recall	In the questions it says, “during the past four weeks”. What are your thoughts on this time frame?	When you are replying to the question, do you think you are able to correctly remember relevant experiences the past four weeks?			Is it an appropriate time frame?		
Length	What are your thoughts on the time it took to complete the questionnaire?						
Format	What are your thoughts on the format?				Was it demanding to fill out the questionnaire?	Did you consider NOT completing the questionnaire?	What suggestions do you have that can make it easier to complete the questionnaire?
Closure	Do you have any last comments?						

The main researcher (MQN) conducted all the interviews. Initially, the interviewer administered the paper version of the MOXFQ to the respondent, asking the respondents to follow the instructions and independently complete the measure. The interviewer registered any facial expressions, body language or verbal cues indicating difficulty completing the form. After completing the questionnaire, the respondents were asked about the comprehension of the wording in the instructions. The interviewer then proceeded to explore their initial thoughts on the questionnaire (“In general, what are your thoughts on the questionnaire?”) [40], and the relevance and understandability of each item based on their experiences after treatment of the ankle fracture (“what are your thoughts on this question? What do you think the question is asking you?” [41]. They were asked about the appropriateness of the recall and response options [41]. Furthermore, they were asked about the length and time it took to complete the MOXFQ, and their thoughts on the questionnaire’s format. Comprehensiveness was assessed by prompting the respondents about other relevant situations pertaining to the domains of the MOXFQ. Additionally, the respondents were asked if there were other topics that they felt were relevant to their experience with an ankle fracture, but not covered by the instrument [42]. Lastly, they were given the opportunity to add additional comments. Interviews lasted on average 37 min (range 18–60). The data sampling was an iterative process, where the data collection and analyses were reviewed by the research team as the sampling progressed. The interviews continued until no new topics were identified following three consecutive interviews.

### Data analysis

In the deductive qualitative analysis, the initial code scheme was based on Tourangeau’s 4-step model of cognitive interviewing [43]. Each step was defined as a category. They were then divided into sub-categories based on the different parts of the questionnaire, i.e., instructions, items, and response categories. Texts that described respondents’ thought processes upon responding on the questionnaire were highlighted as meaning units. The meaning units were condensed and labeled with a code. The codes were sorted according to the appropriate predetermined categories. The labeling and categorization of codes was performed consecutively with the interviews, and the findings were discussed in the review team. The interviews were audio-recorded with a digital voice recorder (Olympus WS-853) and transcribed verbatim using NVivo by the main researcher.

## Results

### Sample characteristics

In total, 17 respondents (65% females) were interviewed (Table 3). Respondents’ age ranged from 27 to 76 years. All respondents had at least 10 years of education. There were office workers and manual workers. Some of the respondents were retired. Respondents had uni- and multi-malleolar fractures. Additionally, one respondent had an open fracture. The fractures were mainly treated with open reduction and internal fixation. One respondent received a fibula nail, and one was treated with syndesmosis screws. The ASA score ranged from one to three. Some of the respondents presented with several comorbidities, e.g., one respondent had diabetes mellitus, hypertension, asthma, and morbid obesity. Another suffered from polyarthritis, hemochromatosis, and hypothyroidism.

**Table 3:** Respondents characteristics (*n* = 17), stratified into three main groups according to recovery phase after surgery. Respondents’ number were given based on the chronological order of the interviews

Recovery phase after surgery	Respondent number	Age group (years)	Gender	Education^a^	Occupation^b^	Fracture characteristics	Treatment	ASA score
Late (longer than 12 months)								
	R1	50–69	Male	Higher education	Teacher	Lateral malleolar	ORIF	2
	R2	70 or older	Female	Higher education	Retired	Tri-malleolar	ORIF	1
	R3	30–49	Female	Lower secondary school	Manual worker	Lateral malleolar	ORIF	2
	R4	30–49	Male	Higher education	Office worker	Proximal fibula and posterior malleolus	Syndesmosis screws	2
	R5	50–69	Female	Upper secondary school	Office worker	Tri-malleolar	External fixation + ORIF	2
Middle (between three and 12 months)								
	R6	70 or older	Female	Higher education	Retired	Tri-malleolar	ORIF	3
	R7	30–49	Male	Upper secondary school	Manual worker	Bi-malleolar	ORIF	2
	R8	50–69	Male	Upper secondary school	Retired	Tri-malleolar	External fixation + ORIF	3
	R9	50–69	Female	Higher education	Retired	Tri-malleolar	ORIF	1
	R10	50–69	Female	Higher education	Teacher	Lateral malleolar	ORIF	2
	R15	70 or older	Male	Upper secondary school	Retired	Bi-malleolar, open	ORIF	3
	R16	70 or older	Female	Lower secondary school	Retired	Tri-malleolar	ORIF	2
	R17	50–69	Female	Upper secondary school	Health-care worker	Bi-malleolar	Fibula nail + screws medially	2
Early (Less than 3 months)								
	R11	30–49	Male	Higher education	Office worker	Lateral malleolar	ORIF	1
	R12	50–69	Female	Higher education	Office worker	Lateral malleolar	ORIF	2
	R13	18–29	Female	Higher education	Teacher	Bi-malleolar	ORIF	1
	R14	70 or older	Female	Upper secondary school	Retired	Bi-malleolar	ORIF	3

^a^The education system in Norway into primary school with seven years of education (ages 6–12 years), lower secondary school with 10 years of education (ages 13–15 years), upper secondary school with more than 10 years of education (ages 16–18 years), and higher education (after secondary school)

^b^Examples of office workers are administrators or engineers. Examples of manual worker are workshop operators or farmers

*ASA* American Society of Anesthesiology (pre-operative physical health status), *ORIF* Open reduction internal fixation

### Comprehension

#### Comprehensibility of instructions

Table 4 summarizes the predetermined categories deducted from Tourangeau’s 4-step model with selected quotations from responders. Most respondents found the instructions brief and easy to understand. Respondent 4 (r4) missed today’s date on the questionnaire, although, he had completed it on the consent form: *“Didn’t read instructions very thoroughly. There were not that many instructions. It was self-explanatory.”* Another respondent (r16) had completed it incorrectly, but correctly on the consent form. There was some confusion regarding instructions on bilateral fractures. This was sorted out once the respondent read the instructions again. See Additional file 3 for additional quotations on each category.

**Table 4:** Predetermined categories deducted from Tourangeau’s 4-step model and selected quotations from responders used in the analyses

Main category	Category	Sub-category	Quotation
Comprehension	Comprehensibility of instructions		*“It was pretty easy to understand what to do. Not that much text and that which was important was emphasized with bold font and underline.”* (r13)
	Feasibility		*“… It’s probably fine, except that I probably should’ve had it in Nynorsk [one of two official Norwegian written languages).”* (r9)
	Comprehensibility of items		*“What is pain then? A little bit? A lot? None? Because when I jumped down there, then it was painful.”* (r3)
Retrieval	Relevance of items	The pain domain	*“Pain in the foot, that I don’t have. I walk freely.”* (r3)
		The walking/standing domain	*“That eight, it is so difficult. I use the car anyways here [chuckles]. We don’t walk when going shopping here.”* (r15)
		The social interaction domain	*“… there are things in the garden that I haven’t managed to do because … it hurts when I’m on my knees … because I get that bend in my foot…”* (r5)
	Incomplete constructs		*“Then you have this issue with vibration in the skin against the plates. It is a bit uncomfortable … Electric scooter on cobblestone [laughs].”* (r7)
			*“If I’ve been sitting for a long time, then it’s painful to start. Then it’s so stiff… That’s probably when it’s most painful.”* (r9)
			*“You’re divorced, as I’ve said, so the kids had to stay with their mom in that entire period.”* (r4)
	Recall		*“Yes, it is okay [to think back on the past four weeks]. Because I feel it has been stable lately.”* (r17)
Judgement	Instrument		*“Today, irrelevant, because I feel that my foot is doing so well. But when I filled it out the last time, then it was probably more relevant … half a year after I broke my foot.”* (r4)
	Domains		*“I couldn’t stand that long. I couldn’t walk that much. I was limping down the hallways. Everybody could see me coming … And that’s why I got the lightest tasks so that I would avoid standing and walking.”* (r7)
	Items		*“… those nerve pains appear, then it just strikes you. But it could also be … that they are mild, the other pain, like an aching-pain sort of … There is sort of a difference between the pains. Those nerve pains are painful. But it doesn’t last that long. Just a few seconds … But it was probably those nerve pains I was thinking of when I crossed off moderate”* (r17)
			*“I don’t think I need to ask about that, really. Because to me it doesn’t mean anything what other might think. Entirely indifferent.”* (r15)
	Missing constructs		*“Yes, sometimes you can feel that the foot is so stiff, you know. That you’re done, just like you have … I was going to say prosthesis, but … Something is not right, but … it was different before I had the fracture.”* (r16)
Response process	Absent response		*“I think it was just that I forgot there, because I was thinking that sometimes … But when I think more about it now, then I see that there are different situations that I have … Well, yes …”* (r5)
	Deduction of information to a suitable response		*“… you added daily activities, so it’s alright then … if it had been just work, then I would have been hesitant to what to answer, because I haven’t been to work, but that has nothing to do with the foot”* (r3)

#### Feasibility

The respondents reported that the questionnaire was easy to understand and didn’t take too long time to fill out. There weren’t any complicated words or phrases. One respondent (r3) commented that item 1 and 15 should be next to each other as these were related: *“But if you take number … question number 1 and put it right above 15, then like “I have pain in my foot. Ehh yes. And how much pain do you have?” Right? It’s a bit like that. So those two should be one after another.”* Another respondent (r9) would like to have the questionnaire in Nynorsk (one of two official Norwegian written languages). Almost all patients did not circle the appropriate side on top of page 2. The reason was that they missed the instructions: *“I don’t know why I didn’t see it. It clearly says …”* (r9).

#### Comprehensibility of the items in MOXFQ

In general, the respondents did not identify any major problems with the language or intent of the items. There were slightly different interpretations of the word *pain.* One respondent (r3) believed this to be a very harsh word, e.g., related to the moment she fractured her ankle. Two other respondents (r2 and r16) denied that they had pain and described it as *discomfort*. Respondents also reflected upon the type of pain, or the situations they experienced it: *“pain is difficult to describe, if it is due to surgery or weightbearing, or just stiffness after you have used it”* (r3).

For item 2, respondents commented that *long distances* were dependent on the person, and measured this in how many hours they could walk. One respondent (r9) was confident that she walked longer than other people at her age and suggested that the items could be worded such that they specified the activity according to the individual. For item 9, which addressed patients’ feeling of self-consciousness regarding their ankle, the general understanding of the question was if they were worried about others opinion about their foot. One different interpretation on the question was if other people cared about them: *“… Others are interested in how I’m doing, and …”* (r17). The word *“usually”* in item 15 caused slight difficulty in one respondent, where she believed the question to be asking about the status before the injury: *“… like usually, as in prior to surgery then? It says the last four weeks. So usually is like in the old days …”* (r3).

### Retrieval of relevant information

#### The pain domain of the MOXFQ

The respondents described various relevant experiences for all items in the pain domain, i.e., item 1, 11, 12, 15, and 16 (Additional file 4). Late-phase recovery respondents reported that the items were relevant, but less now when compared to earlier in the recovery stage since they were less bothered by pain now. The patients that had the surgery less than one year ago, i.e., early- and middle-phase recovery respondents, found the items more relevant. For instance, on item 1: *“Yes, I’ve experienced that sometimes. However, mild and brief”* (r13). Similar descriptions were also reported for item 15. The respondents found item 11 highly relevant, but usually they believed this pain to be related to the level of exertion during the day: *“My foot is worse in the evening, depending on what I’ve done during the day. Like yesterday, two hours of padel [a racket sport that combines tennis and squash], then I had to limp to the bathroom later”* (r7). The two remaining items in this domain, i.e., item 12 and 16, the respondents had more diverse experience as not all respondents reported that they had experienced shooting pain or had trouble sleeping: *“Sometimes when I’ve gone to bed … Sometimes I wonder if it is the screws that … When I’m laying sideways, that the screws somehow … That I feel them”* (r9).

In general, the respondents believed that the construct *pain* was covered by the items in the pain domain. They experienced other bodily pain when asked about additional relevant experiences with pain, e.g., pain in the thigh and hip when rolling over in bed due to the cast. They also mentioned stiffness and pain due to swelling of the foot and ankle, or that the swelling led to pain when wearing shoes: *“Stiffness. I can feel it here. It’s just like there’s a band attached here and it’s tightening.”* (r15).

#### The walking/standing domain of the MOXFQ

Respondents that were interviewed shortly after surgery indicated problems recalling relevant episodes for the walking/standing domain, i.e., item 2 to 8, as they were not allowed weightbearing yet. Respondents from later recovery phases were more likely to share relevant experiences for items in this domain (Additional file 5): *“I’ve walked downtown to [city], and then walked around there for a bit. Of course, you would always feel some discomfort, but not like it’s the end of the world!”* (r16). Item 8, asking about the use of bus or car, had less relevance for people living on the countryside since they were dependent on using the car for everyday tasks in the first place.

When asked about other relevant limitations related to mobility, respondents commented that they experienced stiffness and pain when they were about to start walking again after resting or sitting still for a while. Additionally, some would feel pain in the other foot when walking long distances or if they have been standing still for a while.

#### The social interaction domain of the MOXFQ

The items included in the social interaction domain (item 9, 10, 13 and 14) were intended to measure limitations in social interaction. Two of these items, item 9 and 10, referred to self-consciousness about their foot/ankle and the use of footwear, respectively. These items emerged as less relevant to the respondents since not all were able to share relevant thoughts or experiences regarding these issues (Additional file 6). Respondent 13 shared her thoughts when interacting with children shortly after surgery, where she was worried about the impression she would leave for her young students if they saw her ankle *“… Lately, the weather has been nice, and you’ve been using summer shoes and summer clothes … when you’re working as a teacher, and I’m on a primary- and lower secondary school, then sometimes I’ve been thinking: “poor kids if they [laughs] … have to look at this half-swollen foot after a day with a lot of walking and stuff”, but …”* (r13).

The respondents were asked to recall other important experiences that had an impact on the social interaction due to the ankle fracture. The burden of being dependent on others was mentioned, e.g., having to leave his children with his divorced wife, or asking a parent to help with grocery shopping. Another example was refraining oneself from going to a festival due to fear of a new injury.

#### Recall

A recall period of four weeks was acceptable for the respondents, except when replying to the survey at early stages since they would experience a considerable improvement within a short time span: *“… there was a huge difference for … at least for me … Four weeks ago, then it was one week after surgery. At that point things were miserable and painful … Now it is sort of … What should one base it on? … Because it says, “during the past 4 weeks” and there is a progression in it … The first two weeks were definitely worst. Then things started to stabilize a bit and …”* (r11). Respondents later in the recovery process felt things were more stable and that it was easier to remember specific and relevant episodes related to their ankle fracture.

### Judgement

#### General aspects of the MOXFQ

In general, respondents reported that the questionnaire was helpful in assessing the respondents’ experiences after the ankle fracture. The respondents in late recovery phase and those that had experienced very little pain and limitations after the injury did not find the questionnaire as relevant: *“… And for me, I just replied “no” to everything because I don’t have pain. So, there’s nothing that limits me because of that … No matter what situation they ask me about here, then it’s not because of pain that prevents me from doing it …”* (r6). The questionnaire was also experienced as less relevant for respondents in early recovery phase. Respondents had difficulties replying to items concerning limitation in mobility related to pain since they were not allowed to put weight on the operated foot, thereby limiting the relevance of the instrument: *“Because all the questions were based on pain. That the pain was the limitation, but I don’t feel that the pain was restricting me because … because I had to use crutches and I couldn’t walk without the crutches and put full weight on my foot, so I never reached the point where it was the pain that hold me back”* (r13).

When asked about the adequacy of the instrument to cover important aspects concerning the impact from the ankle fracture, respondents pointed out the lack of questions asking about psychological well-being, worrying thoughts regarding surgery and the complications from surgery, or *“the feeling of uselessness”* (r15). One patient summed it up: *“… So here, it’s like pain and discomfort, but it doesn’t really ask about how I’m really doing”* (r7). Beyond the questions in the MOXFQ, respondents experienced other physical symptoms, e.g., stiffness, swelling or heaviness in the foot.

#### Domains of the MOXFQ

The assessment of pain was difficult for some respondents. For instance, when late-phase recovery respondents were asked about the relevance of pain related to their ankle fracture, they would reveal that they didn’t have pain, but perhaps discomfort at certain situations: *“For me pain is … then it hurts in a way. I don’t have it. I might feel discomfort … Great pain, that’s when I broke my ankle and could feel the vomit. Then it was painful …”* (r3). Others would think that *“the pain that I had was probably related to surgery”* (r1). One respondent from the middle recovery phase reported how the ankle fracture affected him at work, e.g., that his inability to stand or walk for longer periods prevented him from doing his normal chores.

#### Items in the MOXFQ

The cognitive effort used to evaluate the relevance of each item was generally feasible, supported by the respondents’ motivation to attempt answering all items. Respondents from the late recovery phase had good scores when examining the replies on the questionnaire, as the question mostly addressed pain and pain-related limitations: *“I replied rarely or none on most of them … That says something about the use of words or … It might be correct, but it is the use of words to describe pain that I have an issue with. I, that don’t have pain …”* (r2). Respondents from this phase would add that they experienced other difficulties, e.g., discomfort or problems fitting normal shoes due to a swollen foot.

Respondents from the middle recovery phase experienced minor problems, although a few also had none to very little problems based on the scoring on the questionnaire (r6 and r10). They experienced pain related to weightbearing, walking in stairs, or after extensive use. One respondent described an alteration in the character of pain with more sudden intense pain now: *“… It’s painful now. Those pains. I didn’t notice that too much during the first half year. But now, this is bothering me. But you sort of get used to it. That “ohh! Now I must change position”, and it appears like POFF!”* (r17). She experienced it every day and was contemplating on *rarely* or *most of the time* as the final response. Additionally, this sudden intense pain was considered most important when replying to item 15, as it was the most prominent pain, though, she did have a milder, constant ache.

Item 9 and 10 were the only items in the questionnaire that were not related to pain. A large number of respondents were not preoccupied with the opinion of others on their ankle or footwear. The males were generally negative to these items and suggestions on removing them were mentioned. An older male respondent was very clear about the irrelevance of this question as he was *“entirely indifferent”* (r15) to what others might think of his foot. However, one respondent disclosed some discerning thoughts about footwear and dresses: *“When you arrive with a nice dress and then … sneakers. But in fact, this is fashion now, now others are doing it, those that didn’t injured their foot … But that’s just now, so I hope that … one day I can wear proper shoes, but I can’t as long as I have those screws there”* (r5). The same respondent also found item 9 relevant to some degree: *“Because you could see pretty well that the ankle was … was much bigger den the other, right? You don’t do that when you have pants on … But if you have a skirt and pantyhose on, then you’ll spot it … So, I’ve thought about it, even though self-conscious might not be the correct term”* (r5).

Item 8, which ask about the need to catch the bus or use a car instead of walking due to pain, caught the attention of some of the respondents living on the countryside. They found it difficult to answer the question as they needed a car to get around independent of the injury. One respondent thought it was slightly odd to be asked such a question and believed this question to be better suited for respondents living in the cities: *“These are questions for people living in [large city], I reckon … Here, I must use a car [chuckles] if you know what I mean … I use a car anyways … Because If I were to go to the store, well, then I would take the car. I don’t bother walking four kilometers back home with …”* (r3).

### Response

Item 7, that asked about the avoidance of standing for a long time due to pain, had one missing response. During the interview, the respondent had to stop and think about it for some time when filling out the questionnaire because *“I don’t stand that much, you know, so I haven’t thought about it”* (r5). She was asked to elaborate on her thought process and the reason for the missed response: *“… because I was thinking of work. But then I don’t stand that much at work. Like when I’ve been to airports, then I’ve avoided standing for longer periods. I would rather go sit down … It has just become a part of me, I think … I think it was just forgetfulness…”* (r5). The same respondent also had some remarks on item 13 since the question addressed limitations in both work and everyday activities due to pain, and she struggled to pick a suitable response option: *“… since I have an office job, it doesn’t prevent me from doing my job. But daily activities … Maybe you should split them?”* Respondent 3 also presented the same reluctancy when replying to this item.

Respondents selected multiple responses on some items. For instance on item 6, which included hard and rough surfaces in the question, the respondent selected one response per adjective describing the surface: *“… hard surfaces I don’t mind. I walk well on asphalt. But if there’s a slope, or especially a steep downhill, then you get this bend and you must sort of limit yourself a little…”* (r15). Another item that had multiple response options selected was item 15. This respondent was still early in the recovery phase and found it difficult to pick one response option due to the use of the word *usually* in the question, with reference to some days the pain would be better than other days: *“When it starts to hurt, then it really hurts”* (r12).

## Discussion

### Main findings

To the best of our knowledge, this is the first qualitative study to explore aspects of relevance, comprehensiveness, and comprehension, i.e., the content validity, of the MOXFQ when applied to an ankle fracture population by investigating the response process. The study utilized a deductive approach to examine if the existing theory concerning life impacts for patients with a hallux valgus deformity could be accommodated to an ankle fracture population. Some crucial aspects were highlighted when applying the MOXFQ to this context. Firstly, pain was an important but difficult concept in the follow up after surgical treatment of ankle fractures and respondents perceived pain differently during the course of recovery. The items that measured pain and pain-related limitations in mobility were relevant for patients that were allowed WBAT. However, the subscale intended to measure limitations in social interactions was perceived as not relevant. Secondly, the index summary score was not comprehensive in measuring the overall impact of ankle fractures on HRQOL. Lastly, the study identified ambiguous wording that should be addressed to improve comprehension and relevance of some items when applied to an ankle fracture population.

### The concept of pain in the recovery phase of ankle fracture patients

The MOXFQ intends to measure two constructs of pain: pain in general, and pain when walking or standing. Pain is well known to have a great impact on a person’s overall quality of life [44] and is often used in the assessment of treatment results, both clinically and in research studies [45, 46]. In the work of developing an instrument to measure overall quality of life by the World Health Organization (WHO) group, six broad domains were identified: physical, psychological, level of independence, social relationships, environment, and spirituality/religion/personal beliefs [47]. In this multi-dimensional construct, *pain and discomfort* were encompassed in the physical domain. However, pain itself is not considered a unidimensional concept. For instance, the McGill Pain Questionnaire assesses quality and intensity of pain [48]. Additionally, patients with chronic pain report poorer overall quality of life compared to patients with acute pain [44]. Therefore, it is understandable that the majority of items in a questionnaire developed to assess a hallux valgus deformity condition consist of questions pertaining to pain. However, the pain in patients suffering from an ankle fracture is of an acute nature. One could argue that patients with these two conditions would experience some of the same challenges and limitations in the recovery phase. For instance, there are over 130 different procedures recommended for the hallux valgus correction [49], but similar post-operative restrictions in weight bearing would apply to both groups. Additionally, the pain experienced by the patients in early stages after the procedure would typically be related to the surgery itself. One would also expect the remission of pain and disability within the first one to two years after surgery [50]. In other words, using MOXFQ for patients in the middle-phase recovery, i.e., between 3 and 12 months after surgery, seems reasonable. In early stages, before WBAT is allowed, one could risk measuring limitations in mobility or in social interaction due to post-operative treatment regimens, and not limitations due to pain as the domains intended. Also, if used earlier than four weeks after surgery, the recall period would include the pre-operative phase, which can be confusing for the respondents. In late phases, ceiling effect (lower scores indicating less disability) could be a potential threat to the instrument’s content validity since patients perhaps no longer associate their main restrictions with pain.

### The constructs measured by the MOXFQ

Overall, the instrument seems capable of capturing key elements of the construct pain and pain when walking or standing. However, the suitability of the items that covered limitations in social interaction was less convincing. Particularly items 9 and 10, where the questions focused on being limited due to people’s view on the appearance of the injured foot or the use of footwear. Although a few would find item 10 more relevant than item 9, e.g., some might experience swelling resulting in the need to use bigger shoes, or prominent osteosyntheses with ensuing discomfort when using hiking boots, the recurrent notions for these items were *“… That’s my business! So that I don’t care about”* (r14). This might be attributed to the development of the instrument, where the instrument’s items were based on patients with a hallux valgus deformity scheduled for surgery, and not an ankle fracture population. A main difference between the two groups is the medical indication for treatment, i.e., hallux valgus correction procedures are mainly performed for the relief of chronic pain, while the treatment of ankle fractures is an emergency or urgent procedure. Another important difference is the sex distribution between the groups. Though, biological factors probably play a role in the development of the hallux valgus deformity, most likely the pain and disability associated with the condition are accentuated by less physiological favorable female footwear, demonstrated by the higher proportion of females undergoing this procedure [51]. In contrast, both sexes would be affected in an ankle fracture population. Additionally, one could not neglect the potential cultural difference: *“… I believe that we have sensible footwear in Norway. Nobody cares if we wear hiking shoes downtown”* (r9).

As 14 of 16 items include pain or pain-related limitations, a summary score labeled the MOXFQ-index was constructed to measure the overall life impact on HRQOL [36]. Where the MOXFQ subscales are clear on the construct they intend to measure, the MOXFQ-index was proposed to measure a more general construct. However, respondents in this study reported the lack of questions addressing other physical symptoms, e.g., swelling and stiffness, and psychological factors, e.g., worrying about complications or fear of falling again. If compared to another study assessing the life impact of ankle fractures [52], the MOXFQ-index lacked constructs to cover the entire aspect of life impacts in patients suffering from ankle fractures, e.g., psychological and financial factors, and medication taking. One could claim that the items of the MOXFQ-index to be relevant in assessing one of the aspects of overall quality of life, i.e., pain-related life impact of ankle fractures. However, the instrument would not be comprehensive in assessing general life impact due to the absence of items addressing other physical and psychological factors. Even if it is simpler to use an index score for the assessment of patients in clinical practice or analyses in a research setting, one should be wary of these limitations when applied to an ankle fracture context.

### Aspects of use in a new population

Some of the items received multiple responses, resulting in uncertainty in the interpretation of the respondents’ answers and complicates the scoring. If two responses have been selected, the user manual for the MOXFQ instructs to select the most severe response option when calculating the scores. Items where multiple responses were selected may indicate some degree of ambiguity in the questions, e.g., item 6 in the walking/standing domain concerning rough and hard surfaces, or item 13 in the social interaction domain. The wording of the latter item addresses both work and everyday activities, leading to discussions on which activity to consider when replying to the question. These questions are referred to as *double-barreled questions* where “respondents must answer two questions with one answer” [53], and should be avoided since they could potentially have adverse effect on validity [54]. A suggestion from one of the respondents was to split the question to enhance the intent of the question. Item 15 from the pain domain faced a similar challenge with the use of the word *usually*. One respondent (r3) from the late recovery phase interpreted the question as if it asked about pre-injury status. Another respondent from early recovery phase explained that the pain varied too much during the last weeks to be able to pick only one option. This reflects one of the challenges when changing the context of use for an instrument, exemplified in this case by applying an instrument intended for patients with stable conditions undergoing elective surgery to patients being treated for emergent or urgent conditions. On the other hand, being too specific in the wording of a question renders it less relevant for many patients, e.g., item 8, which focuses on the need to use secondary transportation methods. The interviews consisted of respondents living in cities and on the countryside, and a repeating voice from the latter group was the need to use a car irrespective of the injury or pain.

The findings of this study entailed patients suffering from an ankle fracture. However, it would be fair to believe that the implications made in this study would also account for patients presenting with similar fractures in the foot or distal tibia undergoing emergent or urgent surgery. Future studies should focus on further validation of the MOXFQ’s measurement properties in a similar context, e.g., validation of the validity, reliability, and responsiveness.

### Limitations and trustworthiness

There are no published studies on the translation and cross-cultural adaptation of the Norwegian version of the MOXFQ. The lack of documentation of the translation and cross-cultural adaption might affect the validity of the instrument.

A sufficient number of respondents (*n* = 17) were purposively sampled to capture the comprehensive voice of this heterogenous group to ensure credibility and transferability of the study to research settings and clinical practice. However, with a deductive content analysis there is a potential bias toward the confirmation of existing theory rather than the disapproval of it since the method is searching for relevant experiences. Respondents were more inclined to share richer descriptions of relevant occurrences compared to thoughts on irrelevant questions, apart from sensitive topics. Additionally, choices were made during the analyses to focus on areas of scarce content, which also could potentially favor the approval of existing theory.

The research team consisted of five researchers with expertise in different fields. The main researcher was a resident orthopedic surgeon and Ph.D. candidate and received guidance from two R.N. Ph.D. professors with extensive experience within this field (AB, MMI). The team also included two consultant orthopedic surgeons with Ph.D. with experience in PROM validation and some experience in qualitative research (AP, KH). The broad expertise of the team contributed to increase the dependability of the study, aiding in decision-making during the analysis process. However, since the main researcher conducted all interviews, there was a risk of introducing bias to the data collection, interpretation, and creation of text due to the orthopedic background. Being aware of this, the interview-guide, transcripts, predetermined categories, and codes were continuously discussed in the research team during the analyses as part of the process to maintain confirmability.

## Conclusion

An ankle fracture population differs from a hallux valgus population in many areas. However, based on the findings of this study, pain and pain-related limitations in mobility are central concepts that remain important to both populations. In conclusion, the content validity of the MOXFQ-pain and walking/standing subscale was acceptable when used in the evaluation of an ankle fracture population that were allowed WBAT. However, the lack of relevance and use of ambiguous language for items covering limitations in social interactions resulted in insufficient content validity for this subscale. Also, the index summary score did not have sufficient content validity due to lack of comprehensiveness in measuring overall impact of ankle fractures on HRQOL.

Future studies should focus on validating the remaining measurement properties of the MOXFQ in a similar context, i.e., the validity, reliability, and responsiveness of the instrument.

### Supplementary Information

**Additional file 1.** The Norwegian version of the MOXFQ. Any use of the MOxFQ in any language and for any purpose can only be made under licence from OUI and by contacting healthoutcomes@innovation.ox.ac.uk. © Oxford University Innovation Limited, 2006. All rights reserved. Norwegian for Norway. Reprinted with permission.

**Additional file 2.** The American Society of Anesthesiologists physical status classification system.

**Additional file 3.** Predetermined categories with selected quotations for each category.

**Additional file 4.** Quotations for the supporting evidence of relevance for the items in the MOXFQ-pain domain.

**Additional file 5.** Quotations for the supporting evidence of relevance for the items in the MOXFQ-walking/standing domain.

**Additional file 6.** Quotations for the supporting evidence of relevance for the items in the MOXFQ-social interaction domain.

## Data Availability

The datasets used and/or analyzed during the current study are available from the corresponding author on reasonable request.

## Abbreviations

ASA: American Society of Anesthesiology
COREQ: Consolidated criteria for reporting qualitative studies
HRQOL: Health-related quality of life
MOXFQ: Manchester–Oxford Foot Questionnaire
PROM: Patient-reported outcome measure
SRQR: Standards for reporting qualitative research
WBAT: Weight-bearing as tolerated
WHO: World Health Organization

## References

[1] Court-Brown CM, Caesar B (2006). Epidemiology of adult fractures: a review. Injury.

[2] Thur CK, Edgren G, Jansson K-Å, Wretenberg P (2012). Epidemiology of adult ankle fractures in Sweden between 1987 and 2004. Acta Orthop.

[3] Daly PJ, Fitzgerald RH, Melton LJ, Llstrup DM (1987). Epidemiology of ankle fractures in Rochester. Minn Acta Orthop Scand.

[4] Elsoe R, Ostgaard SE, Larsen P (2018). Population-based epidemiology of 9767 ankle fractures. Foot Ankle Surg.

[5] Rydberg EM, Wennergren D, Stigevall C, Ekelund J, Möller M (2023). Epidemiology of more than 50,000 ankle fractures in the Swedish Fracture Register during a period of 10 years. J Orthop Surg Res.

[6] Scheer RC, Newman JM, Zhou JJ, Oommen AJ, Naziri Q, Shah NV (2020). Ankle fracture epidemiology in the united states: patient-related trends and mechanisms of injury. J Foot Ankle Surg.

[7] Mehta SS, Rees K, Cutler L, Mangwani J (2014). Understanding risks and complications in the management of ankle fractures. Indian J Orthop.

[8] Verhage SM, Schipper IB, Hoogendoorn JM (2015). Long-term functional and radiographic outcomes in 243 operated ankle fractures. J Foot Ankle Res.

[9] So E, Rushing CJ, Simon JE, Goss DA, Prissel MA, Berlet GC (2020). Association between bone mineral density and elderly ankle fractures: a systematic review and meta-analysis. J Foot Ankle Surg.

[10] Churruca K, Pomare C, Ellis LA, Long JC, Henderson SB, Murphy LED (2021). Patient-reported outcome measures (PROMs): a review of generic and condition-specific measures and a discussion of trends and issues. Health Expect.

[11] Rolfson O, Bohm E, Franklin P, Lyman S, Denissen G, Dawson J (2016). Patient-reported outcome measures in arthroplasty registries. Acta Orthop.

[12] US Department of Health and Human Services (USDHHS). Guidance for industry. Patient-Reported Outcome Measures: Use in Medical Product Development to Support Labeling Claims; 2009. 10.1186/1477-7525-4-79 PMC1629006 17034633

[13] Bernstein DN, Baumhauer JF (2023). Operationalizing PROMs at the musculoskeletal practice and policy levels. J Am Acad Orthop Surg.

[14] Dawson J, Coffey J, Doll H, Lavis G, Cooke P, Herron M, Jenkinson C (2006). A patient-based questionnaire to assess outcomes of foot surgery: validation in the context of surgery for hallux valgus. Qual Life Res.

[15] Dawson J, Boller I, Doll H, Lavis G, Sharp R, Cooke P, Jenkinson C (2011). The MOXFQ patient-reported questionnaire: assessment of data quality, reliability and validity in relation to foot and ankle surgery. Foot.

[16] Norwegian Foot and Ankle Society (NOFAF). Announcement to all members of NOFAF 2015 [updated 14.04.15. NOFAF (Norwegian Foot and Ankle Society) Facebook page]. Available from: https://www.facebook.com/fotankelnorge/

[17] Grün W, Molund M, Nilsen F, Stødle AH (2020). Results after percutaneous and arthroscopically assisted osteosynthesis of calcaneal fractures. Foot Ankle Int.

[18] Stake IK, Ræder BW, Gregersen MG, Molund M, Wang J, Madsen JE, Husebye EE (2023). Higher complication rate after nail compared with plate fixation of ankle fractures in patients aged 60 years or older: a prospective, randomized controlled trial. Bone Jt J.

[19] Husebye EE, Stødle AH (2022). Arthroscopic repair of chronic plantar plate tears of the first metatarsophalangeal joint: a new surgical technique with patient outcomes. Orthop J Sports Med.

[20] Ræder BW, Stake IK, Madsen JE, Frihagen F, Jacobsen SB, Andersen MR, Figved W (2020). Randomized trial comparing suture button with single 3.5 mm syndesmotic screw for ankle syndesmosis injury: similar results at 2 years. Acta Orthop.

[21] Sundet M, Johnsen E, Eikvar KH, Eriksen ML (2021). Retrograde nailing, trabecular metal implant and use of bone marrow aspirate concentrate after failed ankle joint replacement. Foot Ankle Surg.

[22] Abdalla I, Robertson AP, Tippett V, Walsh TP, Platt SR (2022). “I’d never have that operation again”—a mixed-methods study on how patients react to adverse outcomes following foot and ankle surgery. J Foot Ankle Res.

[23] Jia Y, Huang H, Gagnier JJ (2017). A systematic review of measurement properties of patient-reported outcome measures for use in patients with foot or ankle diseases. Qual Life Res.

[24] McKeown R, Rabiu AR, Ellard DR, Kearney RS (2019). Primary outcome measures used in interventional trials for ankle fractures: a systematic review. BMC Musculoskelet Disord.

[25] Nguyen MQ, Dalen I, Iversen MM, Harboe K, Paulsen A. Ankle fractures: a systematic review of patient-reported outcome measures and their measurement properties. Qual Life Res. 2022. 10.1007/s11136-022-03166-3 PMC9829578 35716224

[26] Terwee CB, Prinsen CAC, Chiarotto A, Westerman MJ, Patrick DL, Alonso J (2018). COSMIN methodology for evaluating the content validity of patient-reported outcome measures: a Delphi study. Qual Life Res.

[27] Johnston BC, Patrick DL, Devji T, Maxwell LJ, Bingham III CO, Beaton D, et al. Chapter 18: Patient-reported outcomes. 2021. In: Cochrane handbook for systematic reviews of interventions version 62 (updated February 2021) [Internet]. Cochrane. Available from: https://training.cochrane.org/handbook

[28] National Research Council (1984). Cognitive aspects of survey methodology: building a bridge between disciplines.

[29] American Educational Research Association. Standards for educational and psychological testing: American Educational Research Association; 2014.

[30] Ryan K, Gannon-Slater N, Culbertson MJ (2012). Improving survey methods with cognitive interviews in small- and medium-scale evaluations. Am J Eval.

[31] O’Brien BC, Harris IB, Beckman TJ, Reed DA, Cook DA (2014). Standards for reporting qualitative research: a synthesis of recommendations. Acad Med.

[32] Tong A, Sainsbury P, Craig J (2007). Consolidated criteria for reporting qualitative research (COREQ): a 32-item checklist for interviews and focus groups. Int J Qual Health Care.

[33] Tourangeau R. Cognitive sciences and survey methods. Cognitive aspects of survey methodology: Building a bridge between disciplines, vol. 15; 1984. pp. 73–100.

[34] Willis GB (2005). Cognitive interviewing: a tool for improving questionnaire design.

[35] Willis GB (2015). The practice of cross-cultural cognitive interviewing. Public Opin Q.

[36] Morley D, Jenkinson C, Doll H, Lavis G, Sharp R, Cooke P, Dawson J (2013). The Manchester–Oxford Foot Questionnaire (MOXFQ): development and validation of a summary index score. Bone Jt Res.

[37] Meinberg EG, Agel J, Roberts CS, Karam MD, Kellam JF (2018). Fracture and Dislocation Classification Compendium-2018. J Orthop Trauma.

[38] Patton MQ (2015). Qualitative research & evaluation methods: integrating theory and practice.

[39] Mayhew D, Mendonca V, Murthy BVS (2019). A review of ASA physical status—historical perspectives and modern developments. Anaesthesia.

[40] Brod M, Tesler LE, Christensen TL (2009). Qualitative research and content validity: developing best practices based on science and experience. Qual Life Res.

[41] Patrick DL, Burke LB, Gwaltney CJ, Leidy NK, Martin ML, Molsen E, Ring L (2011). Content validity—establishing and reporting the evidence in newly developed patient-reported outcomes (PRO) instruments for medical product evaluation: ISPOR PRO good research practices task force report: part 2—assessing respondent understanding. Value Health.

[42] Rothman M, Burke L, Erickson P, Leidy NK, Patrick DL, Petrie CD (2009). Use of existing patient-reported outcome (PRO) instruments and their modification: the ISPOR good research practices for evaluating and documenting content validity for the use of existing instruments and their modification PRO task force report. Value Health.

[43] Hsieh H-F, Shannon SE (2005). Three approaches to qualitative content. Analysis.

[44] Skevington SM (1998). Investigating the relationship between pain and discomfort and quality of life, using the WHOQOL. Pain.

[45] Pilskog K, Gote TB, Odland HEJ, Fjeldsgaard KA, Dale H, Inderhaug E, Fevang JM (2021). Traditional approach versus posterior approach for ankle fractures involving the posterior malleolus. Foot Ankle Int.

[46] Herber V, Labmayr V, Sommer NG, Marek R, Wittig U, Leithner A (2022). Can hardware removal be avoided using bioresorbable Mg–Zn–Ca screws after medial malleolar fracture fixation? Mid-term results of a first-in-human study. Injury.

[47] The WHOQOL Group (1995). The World Health Organization quality of life assessment (WHOQOL): Position paper from the World Health Organization. Soc Sci Med.

[48] Melzack R (1975). The McGill pain questionnaire: major properties and scoring methods. Pain.

[49] Azar FM, Canale ST, Beaty JH (2020). Campbell's operative orthopaedics.

[50] Lash N, Horne G, Fielden J, Devane P (2002). Ankle fractures: functional and lifestyle outcomes at 2 years. ANZ J Surg.

[51] Menz HB, Roddy E, Marshall M, Thomas MJ, Rathod T, Peat GM, Croft PR (2016). Epidemiology of shoe wearing patterns over time in older women: associations with foot pain and hallux valgus. J Gerontol A Biol Sci Med Sci.

[52] McPhail SM, Dunstan J, Canning J, Haines TP (2012). Life impact of ankle fractures: qualitative analysis of patient and clinician experiences. BMC Musculoskelet Disord.

[53] Bradburn NM, Sudman S, Wansink B (2004). Asking questions: the definitive guide to questionnaire design–for market research, political polls, and social and health questionnaires.

[54] Menold N (2020). Double barreled questions: an analysis of the similarity of elements and effects on measurement quality. J Off Stat.

