# Supplementary material for: Assessing the content validity of the Manchester–Oxford Foot Questionnaire in surgically treated ankle fracture patients: a qualitative study
Source: J Orthop Surg Res. 2023 Dec 8;18:941. doi: 10.1186/s13018-023-04418-9 (PMC10704649; doi:10.1186/s13018-023-04418-9)
Supplement: Supplementary file 1 — Additional file 1. The Norwegian version of the MOXFQ. Any use of the MOxFQ in any language and for any purpose can only be made under licence from OUI and by contacting healthoutcomes@innovation.ox.ac.uk. © Oxford University Innovation Limited, 2006. All rights reserved. Norwegian for Norway. Reprinted with permission. [file 13018_2023_4418_MOESM1_ESM.pdf]

Manchester-Oxford Foot Questionnaire (MOxFQ)

Norsk versjon (Bokmål) for Norge

Fyll ut følgende før du fyller ut spørreskjemaet:

Dagens dato:

D	D	M	M	Å	Å

På hvilken side av kroppen har du det leddet som du **får eller har fått behandling for**:

Venstre ☐

Høyre ☐

Begge ☐

Hvis du svarte "begge", fyller du ut det <u>første</u> skjemaet med tanke på <u>høyre side</u>. Deretter følger et nytt spørreskjema for venstre side.

© Isis Innovation Limited, 2006. All rights reserved.

Sett ring rundt riktig side **Høyre / Venstre**

Sett et kryss (✓) for hvert utsagn

1. I løpet av de 4 siste ukene har jeg opplevd følgende:
Jeg har smerter i foten/ ankelen min.

Aldri	Sjelden	Noen ganger	Det meste av tiden	Hele tiden
☐	☐	☐	☐	☐

2. I løpet av de 4 siste ukene har jeg opplevd følgende:
Jeg unngår å gå lange distanser på grunn av smerter i foten/ankelen

Aldri	Sjelden	Noen ganger	Det meste av tiden	Hele tiden
☐	☐	☐	☐	☐

3. I løpet av de 4 siste ukene har jeg opplevd følgende:
Jeg endrer måten å gå på, på grunn av smerter i foten/ ankelen

Aldri	Sjelden	Noen ganger	Det meste av tiden	Hele tiden
☐	☐	☐	☐	☐

4. I løpet av de 4 siste ukene har jeg opplevd følgende:
Jeg går sakte på grunn av smerter i foten/ ankelen

Aldri	Sjelden	Noen ganger	Det meste av tiden	Hele tiden
☐	☐	☐	☐	☐

5. I løpet av de 4 siste ukene har jeg opplevd følgende:
Jeg må stoppe og hvile foten/ankelen på grunn av smerter

Aldri	Sjelden	Noen ganger	Det meste av tiden	Hele tiden
☐	☐	☐	☐	☐

6. I løpet av de 4 siste ukene har jeg opplevd følgende:
Jeg unngår enkelte harde og ujevne underlag på grunn av smerter i foten/ankelen

Aldri	Sjelden	Noen ganger	Det meste av tiden	Hele tiden
☐	☐	☐	☐	☐

© Isis Innovation Limited, 2006. All rights reserved.

7. I løpet av de 4 siste ukene har jeg opplevd følgende:
Jeg unngår å stå lenge på grunn av smerter i foten/ankelen

Aldri	Sjelden	Noen ganger	Det meste av tiden	Hele tiden
☐	☐	☐	☐	☐

8. I løpet av de 4 siste ukene har jeg opplevd følgende:
Jeg bruker buss eller bil i stedet for å gå på grunn av smerter i foten/ankelen

Aldri	Sjelden	Noen ganger	Det meste av tiden	Hele tiden
☐	☐	☐	☐	☐

9. I løpet av de 4 siste ukene har jeg opplevd følgende:
Jeg bekymrer meg for hva andre tenker om min fot/ankel.

Aldri	Sjelden	Noen ganger	Det meste av tiden	Hele tiden
☐	☐	☐	☐	☐

10. I løpet av de 4 siste ukene har jeg opplevd følgende:
Jeg bekymrer meg for hva andre tenker om fottøyet jeg er nødt til å bruke.

Aldri	Sjelden	Noen ganger	Det meste av tiden	Hele tiden
☐	☐	☐	☐	☐

11. I løpet av de 4 siste ukene har jeg opplevd følgende:
Smerten i foten/ankelen min er verre om kvelden

Aldri	Sjelden	Noen ganger	Det meste av tiden	Hele tiden
☐	☐	☐	☐	☐

12. I løpet av de 4 siste ukene har jeg opplevd følgende:
Jeg får lynende smerter i foten/ ankelen min

Aldri	Sjelden	Noen ganger	Det meste av tiden	Hele tiden
☐	☐	☐	☐	☐

© Isis Innovation Limited, 2006. All rights reserved.

13. I løpet av de 4 siste ukene har jeg opplevd følgende:
Smertene i foten/ ankelen min forhindrer meg fra å utføre jobben min/ daglige gjøremål

Aldri	Sjelden	Noen ganger	Det meste av tiden	Hele tiden
☐	☐	☐	☐	☐

14. I løpet av de 4 siste ukene har jeg opplevd følgende:
Jeg er ikke i stand til å gjennomføre alle mine sosiale aktiviteter eller fritidsaktiviteter på grunn av smerter i foten/ankelen min.

Aldri	Sjelden	Noen ganger	Det meste av tiden	Hele tiden
☐	☐	☐	☐	☐

15. I løpet av de 4 siste ukene..
Hvordan vil du beskrive smerten du vanligvis kjenner i foten/ ankelen?

Ingen	Svært mild	Mild	Moderat	Sterk
☐	☐	☐	☐	☐

16. I løpet av de 4 siste ukene..
Har du blitt forstyrret av smerte fra foten/ ankelen etter at du har lagt deg om kvelden?

Ingen netter	Bare 1 eller 2 netter	Noen netter	De fleste nettene	Hver natt
☐	☐	☐	☐	☐

Til slutt, sjekk at du har svart på alle spørsmålene.
Tusen takk!

© Isis Innovation Limited, 2006. All rights reserved.
