# Supplementary material for: Assessing the content validity of the Manchester–Oxford Foot Questionnaire in surgically treated ankle fracture patients: a qualitative study
Source: J Orthop Surg Res. 2023 Dec 8;18:941. doi: 10.1186/s13018-023-04418-9 (PMC10704649; doi:10.1186/s13018-023-04418-9)
Supplement: Supplementary file 2 — Additional file 2. The American Society of Anesthesiologists physical status classification system. [file 13018_2023_4418_MOESM2_ESM.pdf]

Additional file 2 The American Society of Anesthesiologists physical status classification system

Classification	Definition
1	A normal healthy patient
2	A patient with mild systemic disease
3	A patient with severe disease
4	A patient with severe systematic disease that is a constant threat to life
5	A moribund patient who is not expected to survive without the operation
6	A declared brain-dead patient whose organs are being removed for donor purposes
