# Supplementary material for: Assessing the content validity of the Manchester–Oxford Foot Questionnaire in surgically treated ankle fracture patients: a qualitative study
Source: J Orthop Surg Res. 2023 Dec 8;18:941. doi: 10.1186/s13018-023-04418-9 (PMC10704649; doi:10.1186/s13018-023-04418-9)
Supplement: Supplementary file 3 — Additional file 3. Predetermined categories with selected quotations for each category. [file 13018_2023_4418_MOESM3_ESM.pdf]

Additional file 2 Predetermined categories with selected quotations for each category

Main category	Category	Sub-category	Quotation
Comprehension	Comprehensibility of instructions		*"Didn't read instructions very thoroughly. There were not that many instructions. It was self-explanatory."* (r4)
			" … I don't know why this is asked about … They ask about it here [refers to the first page] … Oh! I see! That was a bit unclear. I rushed … I rushed past it." (r7)
	Feasibility		*"No, it was fine. Very quickly. It wasn't anything … I have the impression that if you were to reply to questions like these then you should answer whatever comes to your mind. Shouldn't sit there and wonder on something. So that was fine."* (r2)
	Comprehensibility of items		*"Pain is difficult to describe, if it is due to surgery or weightbearing, or just stiffness after you've used it."* (r3)
			"… like usually, as in prior to surgery then? It says the last four weeks. So usually is like in the old days … " (r3)
			"… Others are interested in how I'm doing, and …" (r17)
Retrieval	Relevance of items	Item 1: Pain	*"Yes, I've experienced that sometimes. However, mild and brief."* (r13)
		Item 2: Avoid walking long distances	*"I've walked downtown to [city], and then walked around there for a bit. Of course, you would always feel some discomfort, but not like it's the end of the world!"* (r16)
		Item 3: Change the way of walking	*"For me, it started to be a bit like Charlie Chaplin."* (r15)
		Item 4: Walk slowly	*"Like my husband, he would probably want to walk faster, however, I … I try to keep up with him, but sometimes I need to ask him to show me some consideration."* (r17)
		Item 5: Stop and rest	*"Sometimes I need to stop, rest my foot and stretch the ankle. Like I've said, I feel that I need to soften it up. And then it gets a bit easier to continue walking."* (r8)
		Item 6: Avoid rough and hard surfaces	*"I try to avoid uneven surfaces. Not uphill or straight … If there are grass banks or rough surfaces, like ditches in the terrain, then I will avoid it."* (r8)

Main category	Category	Sub-category	Quotation
		Item 7: Avoid standing long time	*"When I think about it … then I know it's not pleasant to stand for a long time. But I have just … I've avoided it, right?"* (r5)
		Item 8: Bus or car	*"I refuse to walk to the store. I rather use an electric scooter."* (r7)
		Item 9: Self-conscious about foot/ankle	*"… Lately, the weather has been nice, and you've been using summer shoes and summer clothes … when you're working as a teacher, and I'm on a primary and lower secondary school, then sometimes I've been thinking: "poor kids if they [laughs] … have to look at this half-swollen foot after a day with a lot of walking and stuff", but …"* (r13)
		Item 10: Self-conscious about footwear	*"It's relevant because many times I've thought of how exhausting it can be to not be able to wear nice shoes or something like that. But I'm not bothered by it."* (r6)
		Item 11: Evening pain	*"My foot is worse in the evening, depending on what I've done during the day. Like yesterday, two hours of padel, then I had to limp to the bathroom later"* (r7)
			"… you felt it the first week after surgery. And of course … Now, you don't have …" (r1)
		Item 12: Shooting pain	*"… those times that I've had it was when I stumbled and had to catch myself with the foot, slippery floors and stuff …"* (r13)
		Item 13: Work or everyday activities	*"You could say that once I started walking, then … to put weight on my hip, then I had pain, so then I was very happy to get ready meals and stuff."* (r6)
		Item 14: Social or recreational activities	*"… past five weeks we haven't spent time together close to what we used to. We often meet at the squash court or go fishing … "* (r11)
		Item 15: Usual pain	*"The few times that I have it, it is very mild"* (r14)
		Item 16: Night pain	*"Sometimes when I've gone to bed … Sometimes I wonder if it this the screws that … When I've laying sideways, that the screws somehow … That I feel them"* (r9)

Main category	Category	Sub-category	Quotation
	Incomplete constructs	Pain	*"Stiffness. I can feel it here. It's just like there's a band attached here and it's tightening."* (r15)
		Pain when walking or standing	*"But it's when you feel that … Unpleasant, like in … And that it gets swollen when you are standing still a lot." (r15)*
		Limitations in social interaction	*"Dependent on my family helping me. As I've said, my mother had to come and buy food for me. Going to the store. At least in the beginning."* (r4)
	Recall		*"… there was a huge difference for … at least for me … Four weeks ago, then it was one week after surgery. At that point things were miserable and painful … Now it is sort of … What should one base it on? … Because it says, "during the past 4 weeks" and there is a progression in it … The first two weeks were definitely worst. Then things started to stabilize a bit and …"* (r11)
Judgement	Instrument		*"… And for me, I just replied "no" to everything because I don't have pain. So, there's nothing that limits me because of that … No matter what situation they ask me about here, then it's not because of pain that prevents me from doing it …"* (r6)
			"Because all the questions were based on pain. That the pain was the limitation, but I don't feel that the pain was restricting me because … because I had to use crutches and I couldn't walk without the crutches and put full weight on my foot, so I never reached the point where it was the pain that hold me back" (r13)
	Domains		*"For me pain is … then it hurts in a way. I don't have it. I might feel discomfort … Great pain, that is when I broke my ankle and could feel the vomit. Then it was painful … "* (r3)
			"The pain that I had was probably related to surgery" (r1)
	Items		*"I replied rarely or none on most of them … That says something about the use of words or … It might be correct, but it is the use of words to describe pain that I have an issue with. I, who doesn't have pain …"* (r2)
			"… It's painful now. Those pains. I didn't notice that too much during the first half year. But now, this is bothering me. But you sort of get used to it. That "ohh! Now I must change position", and it appears like POFF!" (r17).

Main category	Category	Sub-category	Quotation
			"When you arrive with a nice dress and then … sneakers. But in fact, this is fashion now, now others are doing it, those that didn't injured their foot … But that's just now, so I hope that … one day I can wear proper shoes, but I can't as long as I have those screws there" (r5)
			"These are questions for people living in [large city], I reckon … Here, I <u>must</u> use a car [chuckles] if you know what I mean … I use a car anyways … Because If I were to go to the store, well, then I would take the car. I don't bother walking four kilometers back home with … " (r3).
	Missing constructs		*"But the psychological part of having the feeling of uselessness…"* (r15)
			"… So here, it's like pain and discomfort, but it doesn't really ask about how I'm really doing" (r7)
Response process	Absent response		*"I don't stand that much, you know, so I haven't thought about it."* (r5)
			"… because I was thinking of work. But then I don't stand that much at work. Like when I've been to airports, then I've avoided standing for longer periods. I would rather go sit down … It has just become a part of me, I think … I think it was just forgetfulness…" (r5)
	Deduction of information to a suitable response		*"… since I have an office job, it doesn't prevent me from doing my job. But daily activities … Maybe you should split them?"* (r5)
			"… hard surfaces I don't mind. I walk well on asphalt. But if there's a slope, or especially a steep downhill, then you get this bend and you must sort of limit yourself a little… " (r15)
			"When it starts to hurt, then it really hurts" (r12)
