# Supplementary material for: Assessing the content validity of the Manchester–Oxford Foot Questionnaire in surgically treated ankle fracture patients: a qualitative study
Source: J Orthop Surg Res. 2023 Dec 8;18:941. doi: 10.1186/s13018-023-04418-9 (PMC10704649; doi:10.1186/s13018-023-04418-9)
Supplement: Supplementary file 4 — Additional file 4. Quotations for the supporting evidence of relevance for the items in the MOXFQ-pain domain. [file 13018_2023_4418_MOESM4_ESM.pdf]

Additional file 3 Quotations for the supporting evidence of relevance for the items in the MOXFQ-pain domain

Item	Quotations
**Early-phase recovery**	
Item	Quotations
1	*"Yes, I've experienced that sometimes. However, mild and brief."* (r13)
11	*"And the swelling and pain can often be a bit worse at night than during the day. But that probably depends on if you have used it the entire day."* (r11)
12	*"… those times that I've had it was when I stumbled and had to catch myself with the foot, slippery floors and stuff …"* (r13)
15	*"The few times that I have it, it is very mild"* (r14)
16	*"In the beginning I could barely sleep, or I fell asleep very late. Slept pretty badly."* (r11)
**Middle-phase recovery**	
Item	Quotations
1	*"… Like, I feel pain, but I still don't sit down …* (r17)
11	*"My foot is worse in the evening, depending on what I've done during the day. Like yesterday, two hours of padel [a racket sport that combines tennis and squash], then I had to limp to the bathroom later"* (r7).
12	*"It is painful now. Those pains [lightning pain]. I did not notice it much the first half a year. But now that is bothering me."* (r17)
15	*"… that they are mild, the other pain, like an aching-pain sort of…"* (r17)
16	*"Sometimes when I've gone to bed … Sometimes I wonder if it is the screws that … When I'm laying sideways, that the screws somehow … That I feel them"* (r9).
**Late-phase recovery**	
Item	Quotations
1	*"… When I sit with my foot like this, and then push down a little bit, then I can feel it." (r5)*
11	*"… you felt it the first week after surgery. And of course … Now, you don't have …"* (r1)
12	*"… but I have not had those lightning pains … But I have felt sometimes, when I have been at the physiotherapist, that he probably touched something …"* (r2)
15	*"Well, I think it was … it was relevant …"* (r5)
16	*"That it gets swollen and … or feel that I am swollen. It gets tight, is probably the correct word. It sort of tightens around [the ankle], but it is not painful"* (r2)
