# Supplementary material for: Assessing the content validity of the Manchester–Oxford Foot Questionnaire in surgically treated ankle fracture patients: a qualitative study
Source: J Orthop Surg Res. 2023 Dec 8;18:941. doi: 10.1186/s13018-023-04418-9 (PMC10704649; doi:10.1186/s13018-023-04418-9)
Supplement: Supplementary file 5 — Additional file 5. Quotations for the supporting evidence of relevance for the items in the MOXFQ-walking/standing domain. [file 13018_2023_4418_MOESM5_ESM.pdf]

Additional file 5 Quotations for the supporting evidence of relevance for the items in the MOXFQ-social interaction domain

**Early-phase recovery**	
Item	Quotations
9	*"… Lately, the weather has been nice, and you've been using summer shoes and summer clothes … when you're working as a teacher, and I'm on a primary and lower secondary school, then sometimes I've been thinking: "poor kids if they [laughs] … have to look at this half-swollen foot after a day with a lot of walking and stuff", but …"* (r13)
10	-
13	*"… that it prevents me from carrying out my work or everyday activities, and I have responded "some of the time". Because it is not as easy as it used to be."* (r11)
14	*"… past five weeks we haven't spent time together close to what we used to. We often meet at the squash court or go fishing … "* (r11)
**Middle-phase recovery**	
Item	Quotations
9	-
10	*"It's relevant because many times I've thought of how exhausting it can be to not be able to wear nice shoes or something like that. But I'm not bothered by it."* (r6)
13	*"You could say that once I started walking, then … to put weight on my hip, then I had pain, so then I was very happy to get ready meals and stuff."* (r6)
14	*"I wish that I could have done a little more, and not been afraid. And that I could have gone dancing without thinking that now I will twist my ankle."* (r17)
**Late-phase recovery**	
Item	Quotations
9	-
10	*"This [question] I have given more thought. When you arrive with a nice dress and then … sneakers … "* (r5)
13	*"… there are things in the garden that I haven't managed to do because … it hurts when I'm on my knees … because I get that bend in my foot…"* (r5)
14	*"Of course, when you have grandchildren, so that [question] was appropriate. And I do feel a little bit restricted when playing football."* (r1)
