# Supplementary material for: Assessing the content validity of the Manchester–Oxford Foot Questionnaire in surgically treated ankle fracture patients: a qualitative study
Source: J Orthop Surg Res. 2023 Dec 8;18:941. doi: 10.1186/s13018-023-04418-9 (PMC10704649; doi:10.1186/s13018-023-04418-9)
Supplement: Supplementary file 6 — Additional file 6. Quotations for the supporting evidence of relevance for the items in the MOXFQ-social interaction domain. [file 13018_2023_4418_MOESM6_ESM.pdf]

Additional file 4 Quotations for the supporting evidence of relevance for the items in the MOXFQ-walking/standing domain

**Early-phase recovery**	
Item	Quotations
2	*"… because it could be that I have twisted it a little bit that I avoid walking..."* (r14)
3	*"It was very true, for a long time. I was limping."* (r10)
4	*"The pace is limited with crutches."* (r13)
5	*"It was that that it was like that for a long time … Sit down a little bit and … Yes."* (r10)
6	*"I think it is relevant to ask about and I have responded "most of the time" because I am always aware of the surface…"* (r14)
7	*"… it became painful in the hip of the healthy foot, because it carries much so much of the weight…"* (r13)
8	*"Yes, the first week took … Then it was … Yes, it is relevant."* (r10)
**Middle-phase recovery**	
Item	Quotations
2	*"I've walked downtown to [city], and then walked around there for a bit. Of course, you would always feel some discomfort, but not like it's the end of the world!"* (r16)
3	*"For me, it started to be a bit like Charlie Chaplin."* (r15)
4	*"Like my husband, he would probably want to walk faster, however, I … I try to keep up with him, but sometimes I need to ask him to show me some consideration."* (r17)
5	*"Sometimes I need to stop, rest my foot and stretch the ankle. Like I've said, I feel that I need to soften it up. And then it gets a bit easier to continue walking."* (r8)
6	*"I try to avoid uneven surfaces. Not uphill or straight … If there are grass banks or rough surfaces, like ditches in the terrain, then I will avoid it."* (r8)
7	*"Standing for a long time, that is not pleasant."* (r15)
8	*"I refuse to walk to the store. I rather use an electric scooter."* (r7)
**Late-phase recovery**	
Item	Quotations
2	*"If I am going to walk longer distances, then I need a size bigger [shoe size], that will fit the foot that is [laughs] …"* (r1)
3	*"I used to [change the way of walking], but I do not do it now."* (r3)
4	*"I walked slowly, really… or slowly … the first half a year."* (r1)
5	*"Yes, I am thinking that even though I rarely do it … stop and rest … I have not done it much, but … but it is still relevant."* (r5)
6	*"… because if I come down from a rough path, then I have problems sometimes.* (r1)
7	*"When I think about it … then I know it's not pleasant to stand for a long time. But I have just … I've avoided it, right?"* (r5)
8	*"Yes, I have answered rarely there. That is because I take the car to work anyways, right? I don't feel that I … that it is INSTEAD of walking … But of course, if I had … had the habit of walking to work or something like that, then I would probably take the car a little bit more during that time, right?"* (r5)
